# Systematic integration of m6A regulators and autophagy-related genes in combination with long non-coding RNAs predicts survival in glioblastoma multiforme

**DOI:** 10.1038/s41598-023-44087-6

**Published:** 2023-10-11

**Authors:** Amit Sharma, Yulu Wang, Fangfang Ge, Peng Chen, Tikam Chand Dakal, Maria Stella Carro, Ingo G. H. Schmidt-Wolf, Jarek Maciaczyk

**Affiliations:** 1 Department of Stereotacitc and Functional Neurosurgery, University Hospital of Bonn, 53127 Bonn, Germany; 2 Department of Integrated Oncology, Center for Integrated Oncology (CIO), University Hospital of Bonn, 53127 Bonn, Germany; 3 Genome and Computational Biology Lab, Department of Biotechnology, Mohanlal Sukhadia University, Udaipur, India; 4 Department of Neurosurgery, Medical Center-University of Freiburg, Faculty of Medicine, University of Freiburg, Breisgau, Germany; 5 Department of Surgical Sciences, Dunedin School of Medicine, University of Otago, Dunedin, 9054 New Zealand

**Keywords:** CNS cancer, Cancer epigenetics

## Abstract

Glioblastoma multiforme (GBM) is probably the only tumor in which a unique epigenetic alteration, namely methylation of the MGMT gene, possesses direct clinical relevance. Now with the emergence of aberrant N6 methyladenosine (m6A) modifications (the most common epigenetic modification of mRNA, closely linked to the autophagy process) in cancer, the epi-transcriptomic landscape of GBM pathobiology has been expanded. Considering this, herein, we systematically analyzed m6A regulators, assessed their correlation with autophagy-related genes (ATG), and established a long non-coding RNAs (lncRNA)-dependent prognostic signature (m6A-autophagy-lncRNAs) for GBM. Our analysis identified a novel signature of five long non-coding RNAs (lncRNAs: *ITGA6-AS1, AC124248.1, NFYC-AS1, AC025171.1,* and *AC005229.3*) associated with survival of GBM patients, and four among them clearly showed cancer-associated potential. We further validated and confirmed the altered expression of two lncRNAs (*AC124248.1, AC005229.3*) in GBM associated clinical samples using RT-PCR. Concerning the prognostic ability, the obtained signature determined high-/low-risk groups in GBM patients and showed sensitivity to anticancer drugs. Collectively, the m6A-autophagy-lncRNAs signature presented in the study is clinically relevant and is the first attempt to systematically predict the potential interaction between the three key determinants (m6A, autophagy, lncRNA) in cancer, particularly in GBM.

## Introduction

Glioblastoma multiforme (GBM) is the most common and aggressive form of primary brain tumor in adults with a poor survival rate after diagnosis. Despite multimodality treatment approaches, the therapy of GBM is complicated, partially owing to the heterogeneous nature of this tumor. In fact, surgical resection followed by radiotherapy and concurrent administration of chemotherapeutic drug temozolomide (TMZ) remains the standard treatment options in GBM^1^. Certainly, GBM research in genomics, epigenomics, and transcriptomics has led to unprecedented insights into potential prognostic and predictive indicators. Specifically, two molecular biomarkers, isocitrate dehydrogenase (IDH) mutations and O6 methylguanine DNA methyltransferase (MGMT) promoter methylation, have been linked to favorable TMZ response and improved patient survival.

In the field of epi transcriptomics, aberrant N6-methyladenosine (m6A) modification (most prevalent epigenetic modification of mRNA) has recently been implicated in GBM pathobiology^2^. A recent study showed that m6A modifications play an important role in the development of the tumor microenvironment, stem cell diversity and complexity in GBM^3^. The authors showed that patients with a low m6A score experienced significant therapeutic advantages and clinical benefit. In another study, a risk signature involving seven m6A RNA methylation regulators was also demonstrated to be not only an independent prognostic marker but also predictive of the clinicopathological features of gliomas^4^. The authors also described that m6A regulators were associated with mesenchymal subtype and susceptibility to TMZ in GBM.

Given the growing evidences that m6A is associated with Wnt signaling^5,6^, while recognizing that Wnt/β-catenin plays an inevitable role in GBM^7–9^, there may be possible mechanisms for their yet to be elucidated interplay in the pathogenesis of GBM. Concerning the functional regulation of m6A, the fine-tuning and interactions between the m6A and long non-coding RNAs (lncRNA) axis have been considered. In particular, emerging evidence suggests that m6A RNA methylation-modified lncRNAs play an important regulatory role in gliomagenesis and malignant progression^10–12^. A very recent study established an m6A-associated lncRNA prognostic signature related to overall survival in GBM and discussed its immunomodulatory effect^13^. Undeniably, GBM is one of the most difficult cancers to treat and key regulators are constantly being sought, autophagy is also emerging as a new therapeutic target in GBM^14,15^. Importantly, m6A RNA methylation has been speculated to alter the expression of essential autophagy-related genes to affect autophagy function^16,17^. Despite being a relatively new area of research, there has been considerable evidence that m6A RNA modification crosstalk with the autophagy processes^18–21^. Though the use of autophagy as an adjunctive therapy in GBM is feasible, the essential relationship between m6A and autophagy is still unclear.

Considering this, herein, we systematically analyzed m6A RNA regulators, assessed their correlation with autophagy-related genes, and established and validated lncRNA-dependent prognostic signature for GBM. To our knowledge, this is the first attempt to systematically predict the potential interaction between the three key determinants (m6A, autophagy, lncRNA) in cancer, particularly in GBM.

## Results

### Establishment of m6A-autophagy related lncRNAs for GBM patients

The gene expression data of 23 m6A genes, 209 autophagy genes, and 14,056 lncRNAs were taken from the The Cancer Genome Atlas Program (TCGA) database. Autophagy-related lncRNAs were confirmed when lncRNAs showed correlation with autophagy genes according to Pearson correlation analysis (|R|> 0.4, p < 0.01). Likewise, m6A-related lncRNAs were identified when they showed correlation with m6A genes using Pearson correlation analysis (|R|> 0.4, p < 0.01). Using this criterion, autophagy-related lncRNAs (n = 3530) and m6A-related lncRNAs (n = 1625) were identified. The lncRNAs belonging to autophagy and m6A were considered simultaneously for further analysis, and finally 1539 m6A-autophagy related lncRNAs were identified.

### Construction of an m6A-autophagy-lncRNAs signature for GBM patients

The above mentioned m6A-autophagy related lncRNAs gene expression data were combined with the survival data of GBM patients, which included survival time and survival status (dead or alive), and only 153 patients who met the criteria were considered further. These patients were randomly classified into a training group and a test group at a ratio of 1:1. Subsequently, the prognostic signature was generated first in the training cohort (n = 77). Initially, univariate Cox regression identified 24 autophagy-m6A-related lncRNAs that correlated with survival (p < 0.05) (Supplementary Fig. 1). Subsequently, the lasso regression method was used to further investigate the survival-related lncRNAs and further narrowed down to 9 m6A autophagy-related lncRNAs. Next, we performed multivariate Cox regression to obtain an m6A-autophagy-lncRNAs signature that includes five lncRNAs (*ITGA6-AS1, AC124248.1, NFYC-AS1, AC025171.1, and AC005229.3*), as shown in Table 1. Interestingly, in all three cohorts (training cohort, test cohort, and total cohort), the gene expression of signature lncRNAs varied significantly between high- and low-risk groups.

**Table 1:** Multivariate Cox regression results.

lncRNA	Coef
ITGA6-AS1	1.979
AC124248.1	0.606
NFYC-AS1	-0.669
AC025171.1	0.676
AC005229.3	1.185

### Confirmation and validation of prognostic ability in the obtained signature

**Figure 1:**
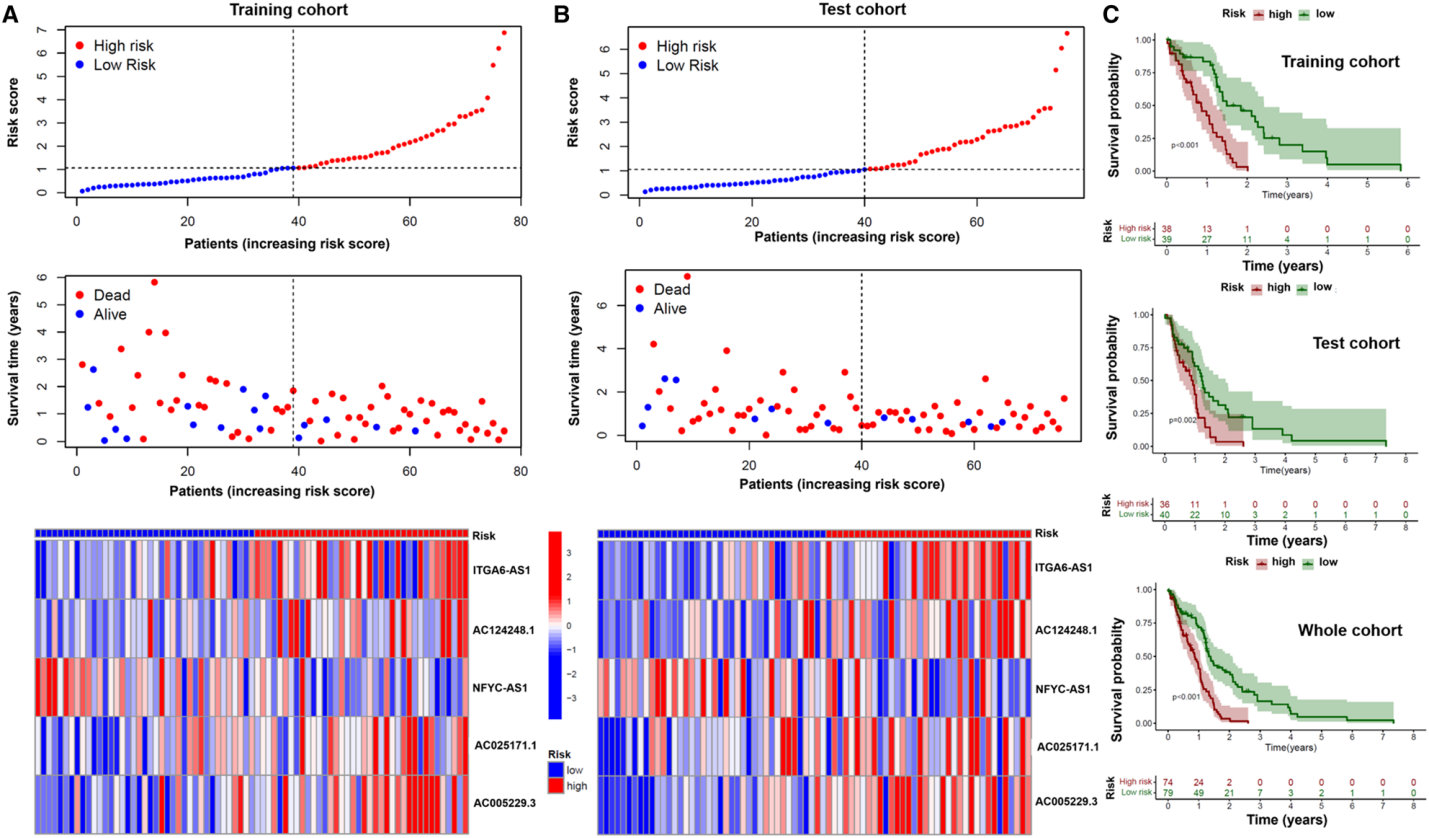
Differential survival time between high and low risk group based on signature in three cohorts. (**A**,**B**) Plot of risk score (upper), plot of survival time (middle) and heatmap (down) between high and low risk group based on signature in training and test cohorts. (**C**) KM (Kaplan Meier) curves for training, test and entire cohorts between high and low risk group based on signature.

**Figure 2:**
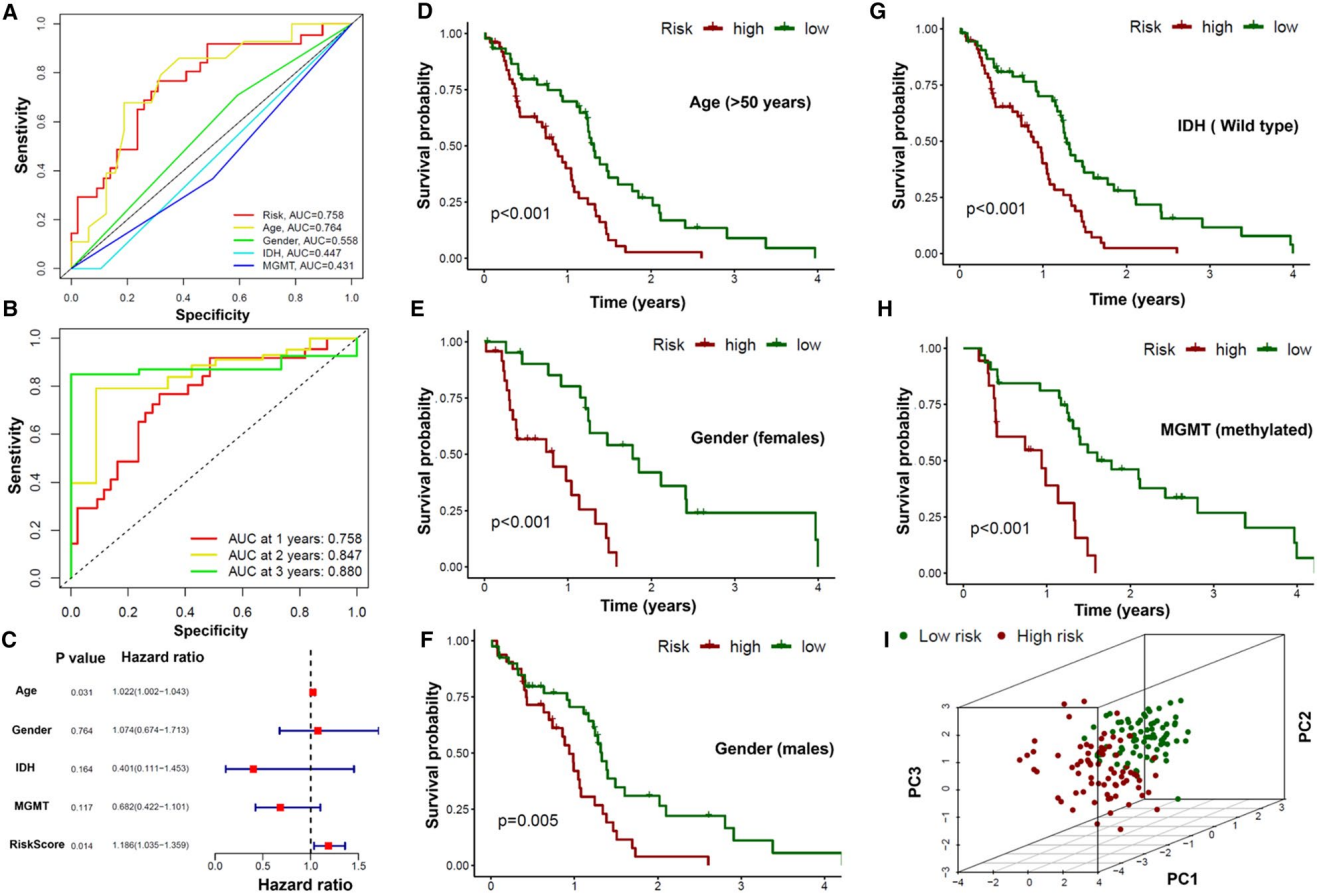
(**A**) Confirmation of survival prediction, differential survival rate in subgroups and PCA analysis based on signature ROC curves for risk score and clinical factors. (**B**) ROC curves for Risk score at 1, 3 and 5 years. (**C**) Mutivariate Cox regression for risk score and clinical factors. (**D**–**H**) KM curves: differential survival in different subgroups between high and low risk group. (**I**) PCA analysis based on the signature. *ROC* receiver operating characteristic, *PCA* principal component analysis, *KM* Kaplan–Meier, *AUC* area under the curve. *IDH* isocitrate dehydrogenase, *MGMT* O6 methylguanine DNA methyltransferase.

The training cohort (n = 77) was used to confirm the prognostic ability of the signature, while the test cohort (n = 76) and the total cohort (n = 153) were used to validate its ability. As shown in Fig. 1, the risk score of high-risk patients was found to be below 1, compared to low-risk patients in the training cohort where it exceeds 1. The percentage of deceased patients in the low-risk group (69.23%) was also found to be lower compared to the high-risk patients (86.84%) in the training cohort. The heat map of the five selective lncRNAs showed the expression pattern between the high- and low-risk groups in the training cohort (Fig. 1A). Interestingly, the test cohort and the total cohort also showed the similar results (Fig. 1B and Supplementary Fig. 2). The risk score of high-risk patients was found to be below 1, compared to low-risk patients in the training cohort where it exceeds 1 in both test cohort and the total cohort. Notably, the percentage of deceased patients in the low-risk group (test cohort: 82.5%; total cohort: 75.95%) was also found to be lower compared to the high-risk patients (test cohort: 88.89%; total cohort: 86.49%). Survival rates were lower in the high-risk group compared to the low-risk group, in the training cohort (p < 0.001), testing cohort (p = 0.002) and in the total cohort (p < 0.001), as shown in Fig. 1C. Gene expression data, survival data, and clinical data were combined for further analysis by including significant number of patients in the training (n = 57), test (n = 61), and total (n = 118) cohorts. We first applied the chi test to test the distribution of clinical data between training and test groups. There were no clinical characteristics and survival status showing unequal distribution between these groups (all p-values > 0.05) as shown in Table 2. Then, receiver operating characteristic (ROC) curves was plotted in the training group, and the results showed that the area under the curve (AUC) was 0.758 (1 year), 0.847 (2 years), and 0.880 (3 years), and the signature risk score had a higher AUC than other clinical characteristics (Fig. 2A,B). Similar results were observed in the other two validation cohorts (test and total cohorts) (Supplementary Fig. 2). To further confirm the prognostic ability of the signature, a C-index (concordance index) analysis was performed in the entire cohort. The concordance index in the risk score of the signature was found to be higher compared with other clinical features. In addition, univariate and multivariate Cox regressions were performed to examine the independent factors (age, risk score), and the signature risk score was found to be an independent factor in predicting survival of GBM patients (Fig. 2C, Supplementary Fig. 2E). We then tested the prognostic ability of the signature in clinical subgroups. The IDH mutation subgroup was excluded because there are no patients with this mutation in the high-risk group. Of note, the high-risk group had lower survival compared to the low-risk group in all clinical subgroups: age < 50 years (p = 0. 026), age > 50 years old (p < 0.001), female (p < 0.001), male (p = 0.005), IDH WT (p < 0.001), MGMT methylated (p < 0.001), and MGMT unmethylated (p = 0.04) (Fig. 2D–H and Supplementary Fig. 2F,G). In addition, principal component analysis (PCA) was performed to investigate the grouping ability between high and low risk patients by using the following parameters: all genes, m6A genes, autophagy genes (ATG), m6A-autophagy-lncRNAs, and five lncRNAs (the obtained signature). As shown in Fig. 2I and Supplementary Fig. 3A–D, only five lncRNAs (the obtained signature) group showed a different distribution between the high- and low-risk groups.

**Table 2:** Distribution of the clinical information and survival status in three cohorts.

Clinical features	Subgroup	Total cohort	Training cohort	Test cohort	P value
Age	≤ 50	23(19.49%)	13(22.81%)	10(16.39%)	0.5181
	> 50	95(80.51%)	44(77.19%)	51(83.61%)	
Gender	Female	46(38.98%)	21(36.84%)	25(40.98%)	0.7856
	Male	72(61.02%)	36(63.16%)	36(59.02%)	
IDH	Mutant	8(6.78%)	4(7.02%)	4(6.56%)	1
	WT	110(93.22%)	53(92.98%)	57(93.44%)	
MGMT	Methylated	50(42.37%)	26(45.61%)	24(39.34%)	0.6154
	Unmethylated	68(57.63%)	31(54.39%)	37(60.66%)	
Survival status	Alive	27(22.88%)	11(18.03%)	16(28.07%)	0.2811
	Dead	91(77.12%)	50(81.97%)	41(71.93%)	

*IDH* isocitrate dehydrogenase, *WT* wild type, *MGMT* O6 methylguanine DNA methyltransferase.

### Evaluation of tumor microenvironment (TME), immune infiltrating cells and immune function based on the obtained signature

The ESTIMATE (Estimation of STromal and Immune cells in MAlignant Tumor tissues using Expression data) algorithm was applied to investigate the correlation of TME with the obtained signature. We found that not only immune scores (p < 0.001) and stromal scores (p < 0.001), but also ESTIMATE scores (p < 0.001) showed higher values in the high-risk group compared to the the low-risk group (Supplementary Fig. 4A). Then, the immune infiltration cells were examined using the CIBERSORT (Cell-type Identification by Estimating Relative Subsets of RNA Transcripts) algorithm and the Wilcoxon rank sum test. The relative proportions of 22 immune cells in each patient and the distribution of these immune cells into risk groups were depicted (Supplementary Fig. 4B,C). Noticeably, the different proportions of the 22 immune cells showed a significant difference between the high- and low-risk groups only for CD8 T cells (Supplementary Fig. 4D). Additionally, immune function was assessed using ssGSEA (single-sample Gene Set Enrichment Analysis), and 11 of 13 immune indicators were found to vary significantly between the high- and low-risk groups (Supplementary Fig. 5). The high-risk group showed upregulation of 11 immune indicators (type II IFN response, APC co-inhibition, parainflammation, HLA, cytolytic activity, pro-inflammatory, T-cell co-inhibition, check-point, T-cell co-stimulation, APC co-stimulation, CCR) compared with the low-risk group.

### Correlating the signature with tumor mutational burden (TMB) and functional analysis

**Figure 3:**
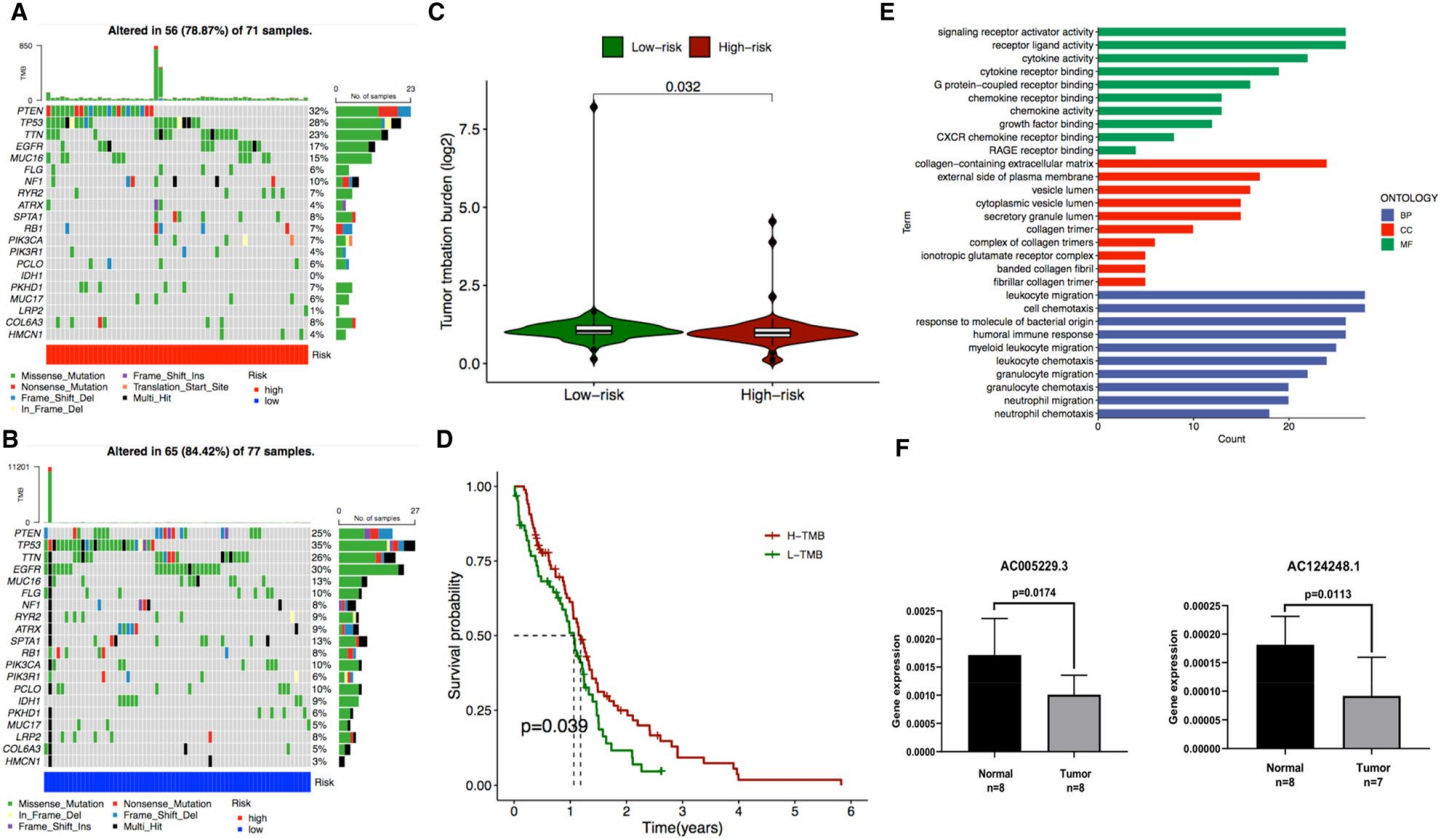
The correlation of signature with tumor mutation burden (TMB) and Gene Ontology Term Enrichment (GO) analysis. Mutation genes in high risk (**A**) and low risk group (**B**). (**C**) Tumor mutation burden in high- and low-risk group. (**D**) Survival rate in high and low TMB group. (**E**) Gene Ontology Term Enrichment (GO) analysis. (**F**) PCR validation in normal ad GBM patient samples.

The R package ‘maftools’ was used to examine the mutation data, and the 20 genes with the highest frequency of change between high- and low-risk groups were identified (Fig. 3A,B). The Wilcoxon rank sum test was applied to evaluate the difference in tumor mutational burden (TMB) values between the high- and low-risk groups, and it was found that the low-risk group had higher TMB values compared to the high-risk group (Fig. 3C). Next, the correlation between TMB and survival in GBM patients was investigated using the KM (Kaplan–Meier) curves. The analysis showed that the group with high TMB had a higher survival rate compared to the group with low TMB (p = 0.039) (Fig. 3D). GO (Gene Ontology) enrichment results showed that the signature is mainly involved immune-related biological processes (BP) in Fig. 3E. KEGG (Kyoto Encyclopedia of Genes and Genomes) analysis showed around signature related 28 pathways (Supplementary Table 1) and present that the signature is involved in immune related pathways. We next validated the obtained signature by performing qPCR using GBM patient samples and confirmed differential expression for *AC124248.1* and *AC005229.3* (Fig. 3F).

### Sensitivity analysis of anti-tumor drugs based on the signature

To further explore the significance of the obtained signature for clinical treatment, we analyzed the sensitivity of GBM patients to all anti-cancer drugs using R package ‘pRRophetic’ and screened out drugs with significant differences in IC50 (half-maximal inhibitory concentration) values between the high- and low-risk groups (P < 0.001). The results showed 23 compounds with different IC50 values among risk groups, with some sensitive to the high-risk group while others to the low-risk group (Supplementary Fig. 6).

## Discussion

Glioblastoma multiforme (GBM) is a devastating form of brain tumor, and despite a number of therapeutic approaches, there have been limited improvements in the clinical scenario. Therefore, it is important to establish prognostic models that can be used for risk stratification and stringent treatment planning. It is also of paramount importance to understand why treatment for GBM is largely ineffective and what clinical/molecular/immunological factors underlie the distinction between short- and long-term survivors. Indeed, two molecular factors, methylation of the promoter of the O6 methylguanine DNA methyltransferase gene (MGMT) and mutation of isocitrate dehydrogenase-1 (IDH1) act as gold standard prognostic indicators. However, owing to the complexity of (epi)genomics, their sole characterization is not sufficient to mark treatment specificity in GBM patients. Of interest, using genomic and clinical databases, several prognostic models have been proposed for GBM patients^22–24^. Among them, prognostic signatures of immune-related genes/lnRNAs^25,26^, epithelial-mesenchymal transition/subtype^27,28^, immune checkpoints/PD-L1-based signatures^29^, and even glioma stem cell signatures^30^, to predict prognosis and response to treatment are of interest.

Since, aberrant regulation of m^6^A mRNA modification has been implicated in many human cancers, including lung, breast liver, gastric, ovarian and pancreatic cancers^31^. Some evidence suggests that m6A alteration may play a key role in gliomas through a variety of mechanisms, providing more opportunities for early diagnosis and targeted therapy of gliomas^2^. Moreover, some m6A based risk score models have also been proposed for predicting the prognosis of glioma patients^32,33^. Recently, m6A methylation regulatory genes have been used to classify patients with low-grade gliomas into high- or low-risk subgroups^34^. Similarly, the m6A-related microRNA risk model has been used as a predictive biomarker for prognosis and immunotherapy in low-grade gliomas^35^. In one study, prognostic analysis and validation of the m6A signature and tumor immune microenvironment was performed in glioma patients^36^. Though the functional regulation of m6A has not been fully elucidated, as mentioned above, some evidence points towards possible interactions between m6A and long non-coding RNAs (lncRNA). As aforementioned also**,** m6A RNA methylation has been speculated to alter the expression of essential autophagy-related genes to affect autophagy function. Therefore, we systematically analyzed m6A RNA regulators and assessed their correlation with autophagy-related genes and established an lnRNA-dependent prognostic signature for GBM. In our comprehensive analysis, we identified m6A-autophagy-lncRNAs signature that includes five lncRNAs (*ITGA6-AS1, AC124248.1, NFYC-AS1, AC025171.1, and AC005229.3*) associated with survival of GBM patients. Of these, four showed cancer-associated potential, e.g., *AC005229.3* and *AC025171.1* have been shown to be a prognostic lncRNA for GBM patients^13^. Also, *AC124248.1* has been associated with the prognosis of other acute myeloid leukemias^37^. Recently, *NFYC-AS1* (Nuclear transcription factor Y subunit C antisense RNA 1) has also been shown to promote lung adenocarcinoma development through autophagy, apoptosis, and the oncogenic proteins MET/c-Myc^38^.

We further investigated the prognostic ability of the obtained signature and demonstrated its suitability in the clinical subgroups such as age, sex, IDH mutation, and MGMT methylation status. Considering that tumor microenvironment (TME) plays an important role in tumor growth and survival, we investigated the correlation between TME and the obtained signature and observed higher immune scores, stromal scores, and ESTIMATE (Estimation of STromal and Immune cells in MAlignant Tumor tissues using Expression data) scores in the high-risk group compared to the low-risk group. The relative proportions of 22 immune cells and the distribution of these immune cells into risk groups were also depicted. Noticeably, the different proportions of the 22 immune cells showed a significant difference between the high- and low-risk groups only for CD8 + T cells. To further extend our analysis, the correlation of tumor mutation burden with the obtained signature was examined, and the results showed that a high tumor mutational burden (TMB) was related to a high-risk group and a low survival rate. Besides immune function, KEGG (Kyoto Encyclopedia of Genes and Genomes) and GO (Gene Ontology) also indicated that the obtained signature is involved in the immune response. As a proof of principle, we validated two lnRNAs (*AC124248.1, AC005229.3*) in the clinical samples using RT-PCR and found alteration in GBM samples compared to the controls. To further explore the significance of the obtained signature for clinical treatment, we analyzed the sensitivity of GBM patients to some anti-cancer drugs and identified 23 compounds with different IC50 values, with some more sensitive to the high-risk group while few to the low risk group.

Collectively, we have established and verified the prognostic signaling model involving m6A-autophgy-lncRNAs in GBM. Our model proves to be clinically useful as both high and low risk groups can be clearly differentiated. It is also worth mentioning the limitations of this study, such as (1) Statistical parameters may have skipped low-expressing lncRNAs in our analysis, and the cumulative effect of those skipped/excluded-lncRNAs might have biological significance. (2) It is unclear whether m6A, autophagy-related genes, and selective lnRNAs target the same PCGs (protein-coding genes)/pathways, so it is of interest whether the preponderance in their interactions has relevant effects at the molecular level.

## Conclusion

We systematically analyzed m6A RNA regulators and assessed their correlation with autophagy-related genes and established a lncRNA-dependent prognostic signature (m6A-autophagy-lncRNAs) for GBM. Our model proves to be clinically relevant as both high and low risk groups can be clearly differentiated.

## Materials and methods

### TCGA data and clinical samples acquisition

Gene expression data (RNA-seq) and mutation data of GBM were retrieved from The Cancer Genome Atlas (TCGA) Program database (https://portal.gdc.cancer.gov/repository, project: TCGA-GBM). A total of 174 samples were available for gene expression, and 13 recurrent samples were excluded from the analysis. Additionally, 389 samples with mutation data were available for analysis. GBM specific survival data (TCGA) were downloaded from UCSC Xena (https://xena.ucsc.edu/, cohort: GDC TCGA Glioblastoma (GBM)). There were 649 samples that included survival data, consisting of information on survival time and survival status. The relevant clinical information was taken from the reported source^39^, 606 samples containing clinical features (age, gender, IDH, MGMT) while only 357 samples were left after removing samples lacking age, gender, IDH and MGMT information. The autophagy related genes information were obtained from the Human Autophagy Database (HADb, http://autophagy.lu/clustering/index.html). The expression data of m6A genes (n = 23), autophagy-related genes (n = 209) and lncRNAs (n = 14,056) were extracted from TCGA gene expression data. Of note, 153 samples (with m6A autophagy lncRNAs gene expression data and survival data), 118 samples (with m6A autophagy lncRNAs gene expression data, survival data and clinical data) and 148 samples (with m6A autophagy lncRNAs gene expression data, survival data and mutation data) were applied in our following computational analysis. For clinical samples, GBM biopsies and cortical samples from epilepsy surgery were collected at the Department of Neurosurgery of the University Medical Center Freiburg (Freiburg, Germany), according to a protocol approved by the Institutional Review Board. Written informed consent was obtained from all participants before their inclusion in the study. An approval for the use of clinical samples was obtained by Dr. Carro from the Etic Commission of the University of Freiburg. All these samples (males: n = 4, tumor grade IV, average age: 62 years; females: n = 4, tumor grade IV, average age: 64 years) used to isolate RNA were clinically well defined. Primarily, the total RNA from tumour samples of patients was prepared in the laboratory of Dr. Carro at the University Hospital Freiburg using the miRNeasy Mini Kit (Qiagen Hilden, Germany) in accordance with the manufacturer's instructions. The RNA concentration was assessed using a NanoDrop 2000 spectrophotometer (Thermo Scientific, Waltham, MA, USA). All the methods (experimental and bioinformatics) were carried out in accordance with relevant guidelines and regulations.

### Construction of prognostic autophagy-m6A-lncRNAs signature

The expression data of autophagy genes, m6A genes and lncRNAs were obtained from 161 samples which excluded 13 recurrent samples from 174 samples. In accordance with the correlation of autophagy genes and lncRNAs, a Pearson correlation analysis was performed to identify the lncRNAs associated with autophagy, termed autophagy-related lncRNAs (|R|> 0.4, p < 0.01). Similarly, m6A-related lncRNAs were confirmed by Pearson correlation analysis on the basis of the correlation between m6A genes and lncRNAs (|R|> 0.4, p < 0.01). Subsequently, the lncRNAs belonging to both autophagy related lncRNAs and m6A-related lncRNAs were determined as m6A-autophagy related lncRNAs (m6A-autophagy-lncRNAs). After combining survival data with data on the expression of m6A-autophagy-lncRNAs, the data from 153 patients/samples were retained. These selected 153 patients/samples were randomly classified into a training cohort and a test cohort at a ratio of 1:1, and univariate Cox regression was performed to find survival-relevant m6A-autophagy-lncRNAs. The output provides information on 24 lncRNAs in the training cohort, and Lasso-Cox regression was applied to further investigate survival-relevant lncRNAs based on tenfold cross-validation and lambda.Min (the lambda value that gives the minimum mean cross-validated error). Then, multivariate Cox regression was used to generate a prognostic m6A-autophagy-lncRNAs signature that comprises 5 lncRNAs, according to the lowest Akaike information criterion (AIC). Having the coefficient of lncRNAs correlated with survival in above multivariate cox regression and expression, the risk score of each patient was calculated as: (βgene 1 × expgene 1) + (βgene 2 × expgene 2) + ⋯ + (βgene n × expgene n). Here, expgene represents the expression of the lncRNA and βgene indicates the coefficient of the lncRNAs. Of note, both the high-risk group and the low-risk group were confirmed based on the cut-off value (median risk score) and the signature was created in the training cohort. In the test cohort and in the total cohort, the risk score was also calculated using above-mentioned formula, the cut-off value is also the cut-off value (median risk score) in training cohort.

### Conformation of the prognostic ability in the obtained signature

KM (Kaplan–Meier) curves and ROC (receiver operating characteristic) curves were applied in training cohorts to confirm the prognostic ability of the signature. Subsequently, the very same analyses were performed in the test and total cohorts to validate the prognostic ability. The concordance index (C-index) was applied in entire cohort to further assess the prognostic ability of the signature. In addition, univariate Cox regression and multivariate Cox regression were performed in the entire cohort to investigate the independent factors for predicting survival of GBM patients. The chi-square test was used to confirm that the baseline clinical data were unbiased between the training, test and entire cohorts. There were 153 samples (with m6A autophagy lncRNAs gene expression data and survival data) used to generate KM curves in this section, while 118 samples (m6A autophagy lncRNAs gene expression data, survival data and clinical data) were used for other analyses.

### Predicting ability of the obtained signature in clinical subgroups

In this particular section, 153 samples with available m6A-autophagy-lncRNAs gene expression data and survival data were analyzed. Clinical features were stratified into subgroups based on age (< 50 vs. age > 50 years old), gender (female vs. male), IDH (wild-type vs. mutated), and MGMT (methylated vs. unmethylated). KM curves were utilized to assess the predictive ability of the obtained signature in each clinical subgroup.

### The correlation between tumor microenvironment and the obtained signature

The ESTIMATE (Estimation of STromal and Immune cells in MAlignant Tumor tissues using Expression data) algorithm was applied to evaluate the correlation between signature and TME (tumor microenvironment). We compared three types of scores (ESTIMATE, immune, and stromal) between high- and low-risk groups based on the obtained signature. In this section, 153 samples (with m6A autophagy lncRNAs gene expression data and survival data) were included in analysis.

### Estimation of immune cells infiltration and immune function in signature

In this section, 153 samples (with m6A autophagy lncRNAs gene expression data and survival data) were included. A CIBERSORT (Cell-type Identification by Estimating Relative Subsets of RNA Transcripts) algorithm was performed to determine the percentage of 22 immune cells in each sample. The Wilcoxon rank sum test was used to compare the difference in immune cell frequency between the high- and low-risk groups by signature (p < 0.05). Of note, the samples in both of the above analyses passed the criterion (CIBERSORT output of p < 0.05) were included in this analysis. In addition, we performed Single-sample GSEA (ssGSEA), an extension of Gene Set Enrichment Analysis (GSEA) and Wilcoxon rank sum test to investigate the difference in immune function between high- and low-risk groups.

### Evaluation of tumor mutation burden (TMB) with signature

Of the 148 samples (with m6A autophagy lncRNAs gene expression data, survival data and mutation data), 77 samples containing mutation information were used in the low-risk group and 71 samples containing mutation information in the high-risk group. The Wilcoxon rank sum test was used to compare the difference in TMB between the high- and low-risk groups. In addition, the difference in survival probability between high and low TMB groups was plotted using the KM curve. The optimal cutoff value for TMB was set using the "surv_cutpoint" function in R.

### Validated of lncRNAs (*AC124248.1*, *AC005229.3*) in clinical samples using RT-PCR

To validate the obtained signature, we selected two lncRNAs (*AC124248.1, AC005229.3*) and confirmed their altered expression in the clinical samples using RT-PCR. cDNA was synthesized using High-Capacity cDNA Reverse Transcription Kit (Applied Biosystems, Waltham, Massachusetts, US). Gene quantification was performed on a QuantStudio3 Real-Time PCR system (Applied Biosystems, Waltham, Massachusetts, US) using PowerTrack SYBR Green Master Mix (Applied Biosystems, Waltham, Massachusetts, US). The cycling program was initiated with 2 min at 95 °C, followed by 40 cycles of 15 s at 95 °C and 60 s at 60 °C. Primer sequences used for validation were:

*AC124248.1*-Fwd: TCCAGAGGATCTGATGGAGC, *AC124248.1*-Rev: CAGTAGCCTGACGCAAATCCT;

*AC005229.3*-Fwd: GCGGCTCATTCCATGAACAA, *AC005229.3*-Rev: GATTTCTGTCCCAGAGCGGT;

*GAPDH*-Fwd: GCACCGTCAAGGCTGAGAAC, *GAPDH*-Rev: TGGTGAAGACGCCAGTGGA. Gene expression levels were normalized to *GAPDH* and 2-ΔΔCt method was used to calculate the relative expression.

### Potentially sensitive drugs for clinical treatment and functional enrichment analysis based on signature

In this section, 153 samples (with m6A autophagy lncRNAs gene expression data and survival data) were included in analysis. The R package 'pRRophetic' was used to predict the IC50 value of compounds from the GDSC (Genomics of Drug Sensitivity in Cancer). The Wilcoxon rank sum test was applied to test the differential sensitivity of drugs in low/high risk group patients according to their IC50 values. KEGG^40^ and GO analyses were performed to find potentially relevant pathways.

### Ethic statement

Patient specimens were used according to approved guidelines (407/09_120965).

### Supplementary Information

Supplementary Information.

## Data Availability

Publicly datasets utilized in this study can be accessed via The Cancer Genome Atlas (https://portal.gdc.cancer.gov/, Project: TCGA-GBM) and Ucsc Xena (https://xenabrowser.net/datapages/, Cohort: GDC TCGA glioblastoma multiforme (GBM).

## References

[1] Li Y, Sharma A, Maciaczyk J, Schmidt-Wolf IG (2022). Recent development in NKT-based immunotherapy of glioblastoma: From bench to bedside. Int. J. Mol. Sci..

[2] Zhang Y (2020). m6A modification in RNA: Biogenesis, functions and roles in gliomas. J. Exp. Clin. Cancer Res..

[3] Wang L, Cao H, Zhong Y, Ji P, Chen F (2022). The role of m6A regulator-mediated methylation modification and tumor microenvironment infiltration in glioblastoma multiforme. Front. Cell Dev. Biol..

[4] Chai R-C (2019). m6A RNA methylation regulators contribute to malignant progression and have clinical prognostic impact in gliomas. Aging.

[5] Kim H, Jang S, Lee Y-S (2022). The m6A (m)-independent role of FTO in regulating WNT signaling pathways. Life Sci. Alliance.

[6] Liu XM, Qian SB (2020). Linking m6A to Wnt signaling. EMBO Rep..

[7] Yusuf S (2022). WNT/β-catenin-mediated resistance to glucose deprivation in glioblastoma stem-like cells. Cancers.

[8] Aretz P (2022). Crosstalk between β-catenin and CCL2 drives migration of monocytes towards glioblastoma cells. Int. J. Mol. Sci..

[9] Koch K (2021). Overexpression of cystine/glutamate antiporter xCT correlates with nutrient flexibility and ZEB1 expression in highly clonogenic glioblastoma stem-like cells (GSCs). Cancers.

[10] Tao N (2022). Interaction between m6A methylation and noncoding RNA in glioma. Cell Death Discov..

[11] Tu Z (2020). N6-methylandenosine-related lncRNAs are potential biomarkers for predicting the overall survival of lower-grade glioma patients. Front. Cell Dev. Biol..

[12] Zheng P, Zhang X, Ren D, Zhang Y (2022). RP11–552D4. 1: A novel m6a-related LncRNA associated with immune status in glioblastoma. Aging.

[13] Xie P (2022). Construction of m6A-related lncRNA prognostic signature model and immunomodulatory effect in glioblastoma multiforme. Front. Oncol..

[14] Sanati M (2022). Recent advances in glioblastoma multiforme therapy: A focus on autophagy regulation. Biomed. Pharmacother..

[15] Batara DCR, Choi M-C, Shin H-U, Kim H, Kim S-H (2021). Friend or foe: Paradoxical roles of autophagy in gliomagenesis. Cells.

[16] Chen X (2021). Current insights into the implications of m6A RNA methylation and autophagy interaction in human diseases. Cell Biosci..

[17] Tang F, Chen L, Gao H, Xiao D, Li X (2022). m6A: An emerging role in programmed cell death. Front. Cell Dev. Biol..

[18] Chen H (2021). The m6A methyltransferase METTL3 regulates autophagy and sensitivity to cisplatin by targeting ATG5 in seminoma. Transl. Androl. Urol..

[19] Chen Y (2021). m6A mRNA methylation regulates testosterone synthesis through modulating autophagy in Leydig cells. Autophagy.

[20] Jin S (2018). m6A RNA modification controls autophagy through upregulating ULK1 protein abundance. Cell Res..

[21] Wang X (2020). m6A mRNA methylation controls autophagy and adipogenesis by targeting Atg5 and Atg7. Autophagy.

[22] Xu H, Zhang L, Xia X, Shao W (2022). Identification of a five-mRNA signature as a novel potential prognostic biomarker for glioblastoma by integrative analysis. Front. Genet..

[23] Jin Y (2022). Comprehensive development and validation of gene signature for predicting survival in patients with glioblastoma. Front. Genet..

[24] Zhang Y (2022). Screening seven hub genes associated with prognosis and immune infiltration in glioblastoma. Front. Genet..

[25] Yu W (2021). Identification of immune-related lncRNA prognostic signature and molecular subtypes for glioblastoma. Front. Immunol..

[26] Wang S, Xu X (2021). An immune-related gene pairs signature for predicting survival in glioblastoma. Front. Oncol..

[27] Ren C, Chang X, Li S, Yan C, Fu X (2023). Epithelial-mesenchymal transition expression profile stratifies human glioma into two distinct tumor-immune subtypes. Brain Sci..

[28] Cheng W-Y, Kandel JJ, Yamashiro DJ, Canoll P, Anastassiou D (2012). A multi-cancer mesenchymal transition gene expression signature is associated with prolonged time to recurrence in glioblastoma. PLoS ONE.

[29] Guo X, Zhang Y, Jiao H, Miao X (2022). The prognostic significance of PD-L1 expression in patients with glioblastoma: A meta-analysis. Front. Oncol..

[30] Chen B (2022). Glioma stem cell signature predicts the prognosis and the response to tumor treating fields treatment. CNS Neurosci. Ther..

[31] Han SH, Choe J (2020). Diverse molecular functions of m6A mRNA modification in cancer. Exp. Mol. Med..

[32] Chen F, Xie X, Chao M, Cao H, Wang L (2022). The potential value of M6a rna methylation in the development of cancers focus on malignant glioma. Front. Immunol..

[33] Luo N (2022). Development of a novel prognostic model of glioblastoma based on m6A-associated immune genes and identification of a new biomarker. Front. Oncol..

[34] Bai Z, Wang X, Zhang Z (2022). Establishment and validation of a 5 M6a rna methylation regulatory gene prognostic model in low-grade glioma. Front. Genet..

[35] Maimaiti A (2022). N6-methyladenosine RNA methylation regulator-related alternative splicing gene signature as prognostic predictor and in immune microenvironment characterization of patients with low-grade glioma. Front. Genet..

[36] Lin S (2020). Prognosis analysis and validation of m6A signature and tumor immune microenvironment in glioma. Front. Oncol..

[37] Zheng G (2022). Comprehensive analysis of N6-methyladenosine-related long noncoding RNA prognosis of acute myeloid leukemia and immune cell infiltration. Front. Genet..

[38] Song Y (2021). LncRNA NFYC-AS1 promotes the development of lung adenocarcinomas through autophagy, apoptosis, and MET/c-Myc oncogenic proteins. Ann. Transl. Med..

[39] Ceccarelli M (2016). Molecular profiling reveals biologically discrete subsets and pathways of progression in diffuse glioma. Cell.

[40] Kanehisa M, Goto S (2000). KEGG: Kyoto encyclopedia of genes and genomes. Nucleic Acids Res..

