# Supplementary material for: Systematic integration of m6A regulators and autophagy-related genes in combination with long non-coding RNAs predicts survival in glioblastoma multiforme
Source: Sci Rep. 2023 Oct 11;13:17232. doi: 10.1038/s41598-023-44087-6 (PMC10567764; doi:10.1038/s41598-023-44087-6)
Supplement: Supplementary file 1 — Supplementary Information. [file 41598_2023_44087_MOESM1_ESM.pdf]

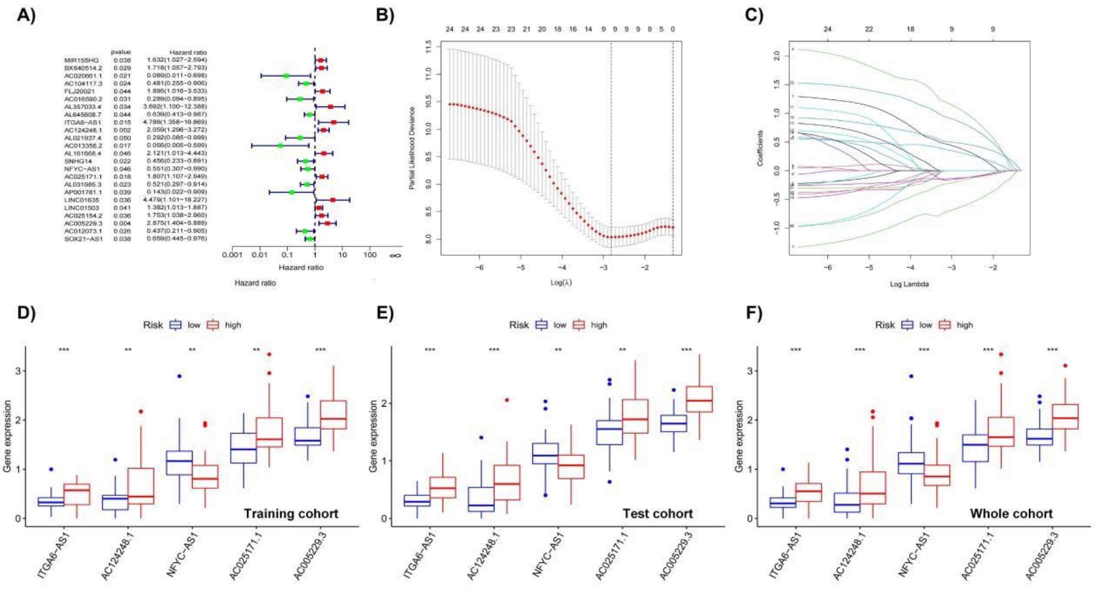

Supplementary figure 1. **Establishment of m6A-autophagy-lncRNAs signature**

A) Univariate Cox regression: survival related m6A-autophagy-lncRNAs. B) 10-fold cross-validation was applied to select best lambda value. Vertical dashed lines represent lambda. Min (left) and lambda. 1SE (right) respectively. C) Based on lambda. Min, the optimal lambda value was applied to select proper survival related lncRNAs. D-F) The differential gene expression of lncRNAs in signature between high and low risk group in different cohorts (training, test and entire cohorts).

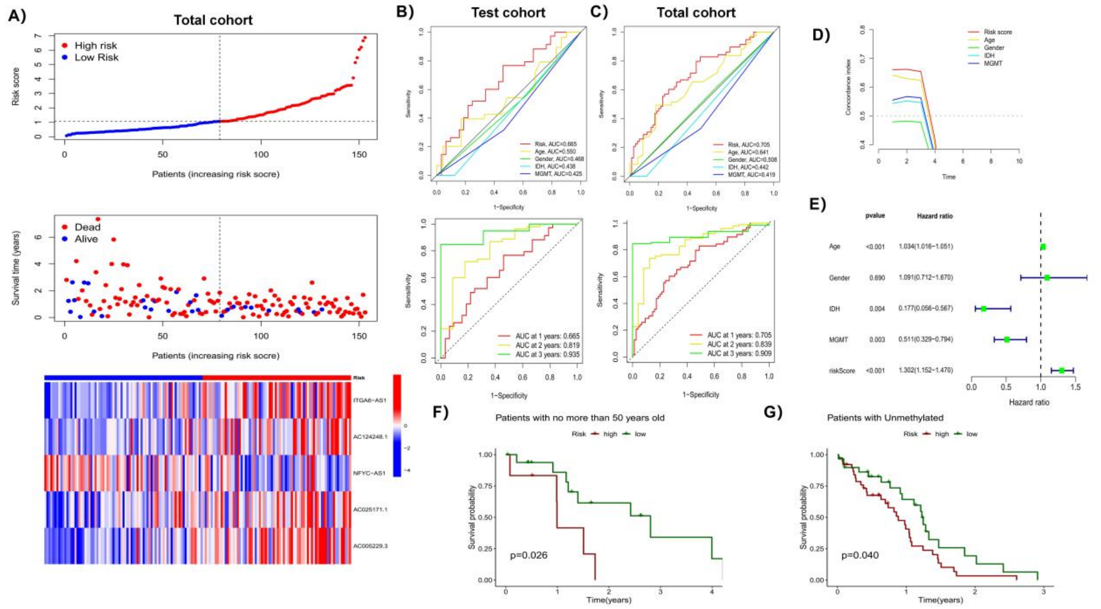

Supplementary figure 2. Confirmation of survival prediction for the obtained signature

A) Survival rate and heatmap. ROC (Receiver Operating Characteristic) curves for test cohort (B) and total cohort (C). D) C-index (Concordance index). E) Univariate Cox regression. F-G) Kaplan-Meier curves

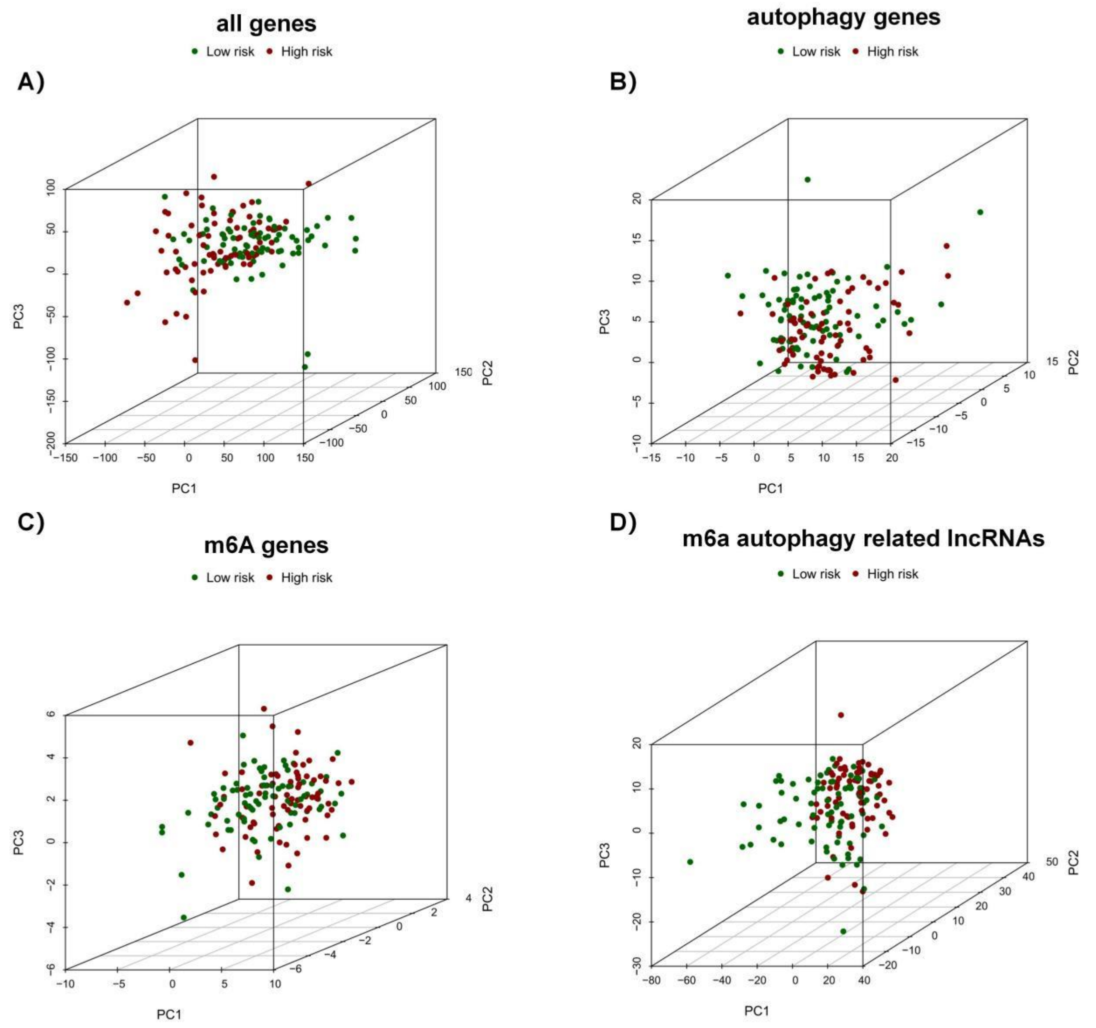

Supplementary figure 3. Principal Component Analysis (PCA)

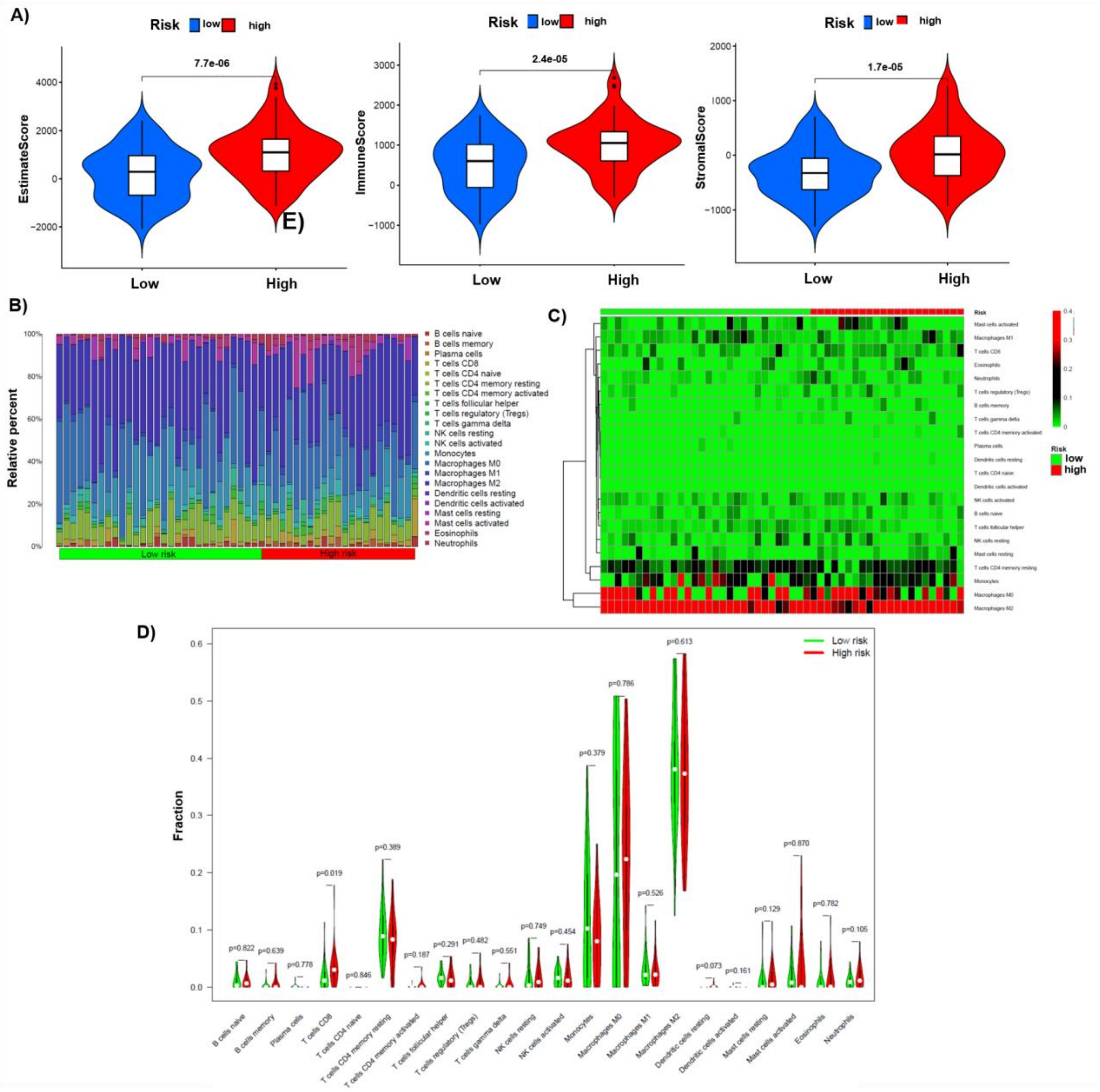

Supplementary figure 4. **Evaluation of tumor microenvironment and immune infiltration cells**

Differential immune score, stromal score and ESTIMATE score between high risk and low risk group. The composition of 22 immune cells (B), heat map (C) and the fractions of immune cells (D) in high- and low-risk group

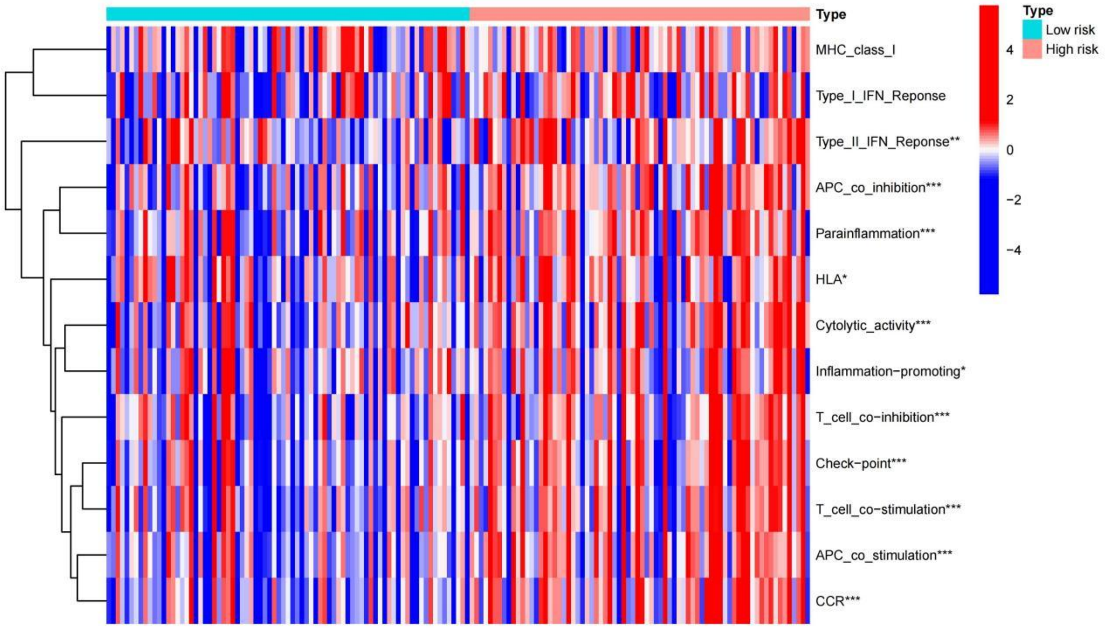

Supplementary figure 5. The alteration of immune indicators based on the obtained signature

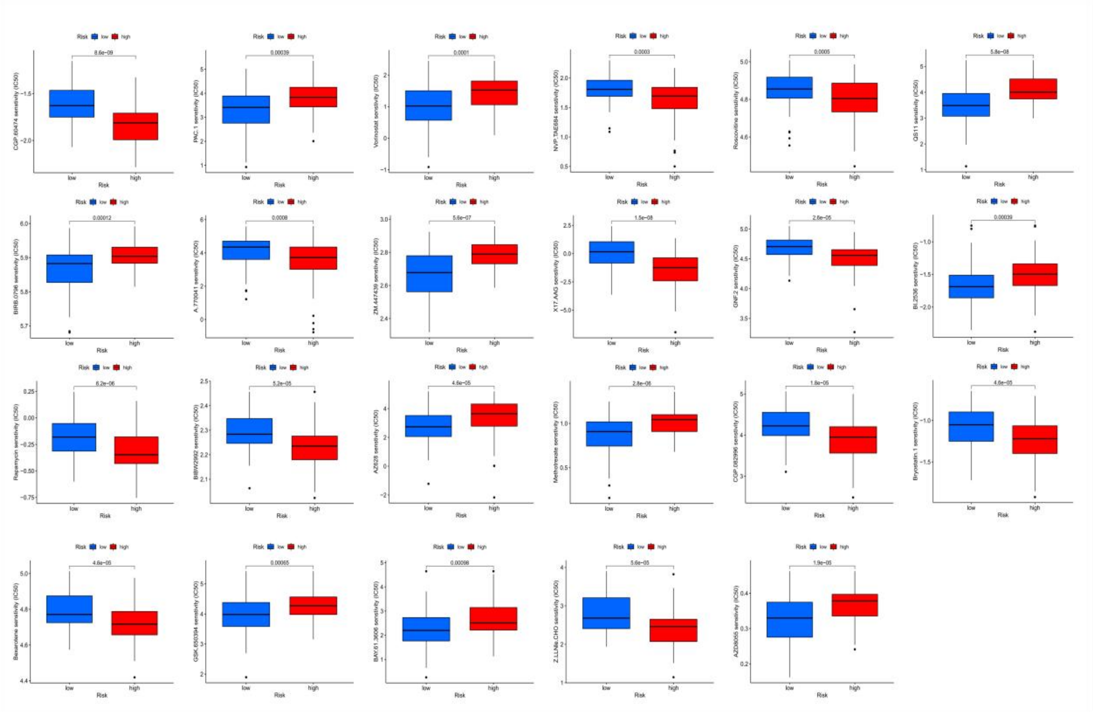

Supplementary figure 6. Differential IC50 value of some compounds between high and low risk groups

Supplementary table 1. Kyoto Encyclopedia of Genes and Genomes (KEGG) enrichment analysis

Pathways	q value
IL-17 signaling pathway	＜0.001
TNF signaling pathway	＜0.001
Viral protein interaction with cytokine and cytokine receptor	＜0.001
Cytokine-cytokine receptor interaction	＜0.001
Rheumatoid arthritis	＜0.001
Amoebiasis	＜0.001
NF-kappa B signaling pathway	＜0.001
Chemokine signaling pathway	＜0.001
AGE-RAGE signaling pathway in diabetic complications	＜0.001
Legionellosis	＜0.001
Protein digestion and absorption	＜0.001
ECM-receptor interaction	＜0.001
Malaria	0.001
NOD-like receptor signaling pathway	0.005
Epithelial cell signaling in Helicobacter pylori infection	0.007
Osteoclast differentiation	0.007
Hematopoietic cell lineage	0.007
PI3K-Akt signaling pathway	0.007
Chagas disease	0.007
Focal adhesion	0.007
Lipid and atherosclerosis	0.011
Complement and coagulation cascades	0.013
Transcriptional misregulation in cancer	0.017
Kaposi sarcoma-associated herpesvirus infection	0.017
Bladder cancer	0.022
Alcoholic liver disease	0.035
Pertussis	0.035
Human cytomegalovirus infection	0.039
